# Quantum Dots Interaction with α-Actinin via Experimental Observations and Computational Predictions

**DOI:** 10.3390/ijms27136070

**Published:** 2026-07-07

**Authors:** Abhishu Chand, Elijah Billue, Tony E. Astuhuaman Davila, Ridwan Sakidja, Kyoungtae Kim

**Affiliations:** 1 Department of Biology, Missouri State University, 901 S National, Springfield, MO 65897, USA; ac43s@missouristate.edu (A.C.); billue345@missouristate.edu (E.B.); 2 Department of Physics, Astronomy, & Materials Science, Missouri State University, 901 S National, Springfield, MO 65897, USA; ta787s@missouristate.edu (T.E.A.D.); ridwansakidja@missouristate.edu (R.S.)

**Keywords:** quantum dots, α-actinin, actin cytoskeleton, physics-based modeling, nanoparticle–protein interactions, cytoskeletal toxicity

## Abstract

Quantum Dots (QDs) are nanoparticles that are highly desirable for biomedical applications such as drug delivery, cellular tracking, and imaging due to their fluorescent and tunable optical properties. However, the biochemical mechanism of their interaction with intracellular proteins that regulate cytoskeletal organization remains poorly understood. While previous studies have shown QDs’ ability to interact with actin and alter actin dynamics, their impacts on actin-binding proteins have not been explored. In this study, we investigated the interaction between CdSe/ZnS QDs and the actin-binding protein, α-actinin, and assessed its impact on actin cytoskeletal organization. Our results demonstrated a strong interaction between QDs and α-actinin, which impeded an α-actinin-mediated filamentous actin (F-actin) bundling, as well as compromised the activity of α-actinin in preventing actin depolymerization. Furthermore, the physics-based modeling and simulations carried out at physiological temperatures supported these findings by identifying stable interaction surfaces between QDs and α-actinin. This study provides mechanistic insight into nanoparticle–protein interactions and highlights the potential cytoskeletal toxicity associated with it.

## 1. Introduction

Drug delivery is an advancing field in biomedicine that aims to increase the therapeutic efficacy. Traditional systems such as tablets, capsules, and various forms of injections use established administration routes [1,2] and are readily available but face challenges in terms of solubility, drug release and distribution, which are all associated with off-target toxicity and limited effectiveness [3,4]. These limitations are critical in cancer therapy, where drugs are unable to reach the diseased cells effectively, thereby causing systemic side effects, including cardiotoxicity and gastrointestinal toxicity [5,6]. As such, modern drug delivery strategies have been explored to overcome these side effects. Among these, quantum dots (QDs) offer solutions by acting as functionalized drug carriers for specific delivery.

QDs are semiconductor nanoparticles that are highly valued for biological applications due to their size and tunable properties. Some common QD types include CdSe/ZnS, CdTe, InP/ZnS, and carbon-based QDs. Each QD is optimized for biological compatibility according to its size, composition, and surface chemistry. Among these, CdSe/ZnS QDs are promising due to their high quantum yield, which enables efficient light emission and real-time observations of biological processes for a long time. Moreover, the ZnS shell can be functionalized for carrying drugs [7].

Given their unique properties, extensive research has focused on using QDs as drug carriers, biosensors, and imaging probes. However, there are major concerns regarding QD-induced cytotoxicity [8,9], and more importantly, their interactions with intracellular proteins remain poorly characterized. QDs non-specifically interact with proteins such as actin, altering the structure of monomeric actin [10] and disrupting actin dynamics [11]. This dynamic assembly and disassembly of actin play an essential role in providing the mechanical support and cellular movement [12]. Subsequently, actin dynamics in cells are regulated not only by actin but also by a network of actin-binding proteins like α-actinin, profilin, cofilin, and tropomyosin [13]. Among them, α-actinin is a key actin crosslinking protein that bundles and stabilizes actin filaments, playing a pivotal role in muscle contraction and cell motility [14]. The effects of QDs on actin in the presence of actin-binding proteins such as α-actinin are unknown and considering the importance of actin-binding proteins in actin dynamics, it is crucial to understand how QDs influence these regulatory components.

In addition to the rise of nanomedicine, computational modeling has become an essential tool in modern drug discovery. Computational tools help bridge the gap between experimental data and theoretical understanding. It provides a cost-effective and scalable approach to not only understand protein structure but also study protein–ligand (QDs) interactions at the atomic level via predicting any structural changes and binding affinities. This physics-based modeling further helps simulate complex physiological environment(s) to build on existing knowledge about how nanoparticle properties influence protein dynamics as well as give predictive insight into nanoparticle behavior in vivo.

Despite the growing interest in the biological applications of QDs, their interaction with actin-binding proteins that regulate cytoskeletal organization remains poorly understood. While previous studies have primarily focused on direct QD–actin interactions, it is unclear whether QDs can interact with regulatory proteins such as α-actinin and affect actin dynamics. Therefore, to our knowledge, this is the first study that examines how CdSe/ZnS QDs interact with α-actinin and how it further influences actin structure and dynamics. We integrated biochemical methodologies with computational approaches to characterize QD–α-actinin interactions and assess their effects on actin organization. This study provides new insights into how QDs disrupt cytoskeletal integrity and cause toxicity.

## 2. Results

### 2.1. QDs’ Characterization

**Figure 1:**
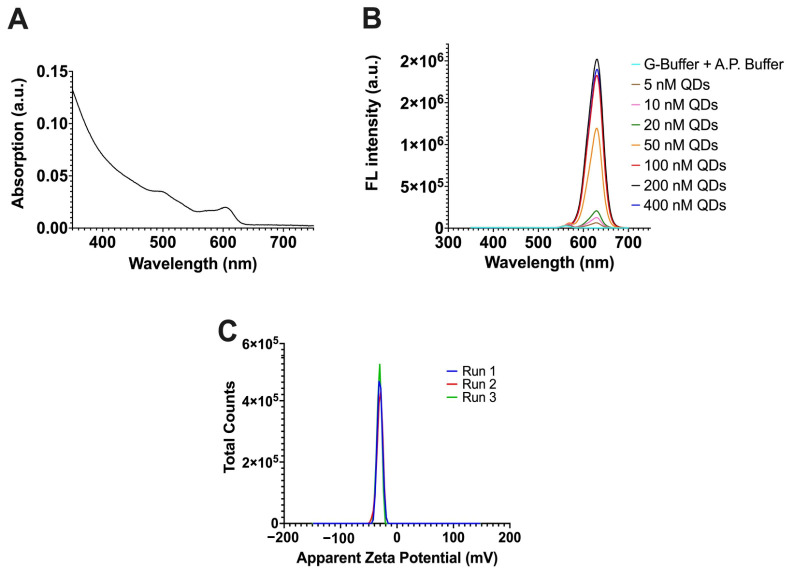
Characterization of CdSe/ZnS QDs. (**A**) Absorbance curve of 50 nM QDs in Millipore water with a scanning range of 350–750 nm. (**B**) Emission spectra of different QD concentrations upon 280 nm excitation. (**C**) Electric potential of 100 nM QDs measured in triplicate.

The QDs have a high absorbance between 350 and 450 nm and show a broadened excitonic peak around 600 nm (Figure 1A). Therefore, it was expected that the QDs should emit red fluorescence, which was also confirmed by our emission spectra (Figure 1B). The broadened peak around 600 nm could be indicative of particle aggregation. Additionally, the absence of a secondary peak suggests that the QDs are relatively stable (Figure 1A). The emission peak of CdSe/ZnS QDs was measured at 630 nm (Figure 1B) with an electric potential of −30.4 ± 4.47 mV (Figure 1C), all of which is consistent with the provided information from the manufacturer, as the QDs are coated with dihydrolipoic acid (DHLA), which binds to the QDs’ surface through thiolate-metal coordination bond. The absence of peaks between 300 and 450 nm (Figure 1B) indicates that QDs do not spectrally overlap with α-actinin’s intrinsic fluorescence, ensuring minimal interference to the α-actinin–QD binding measurement described in Section 2.2.

**Figure 2:**
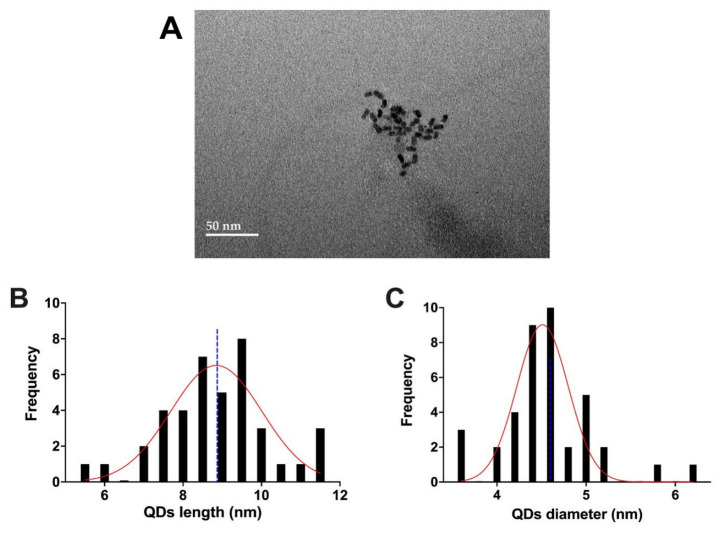
QDs’ characterization using transmission electron microscopy (TEM). (**A**) TEM image of CdSe/ZnS QDs in water, with a scale bar of 50 nm. (**B**) Frequency distribution of QD length. (**C**) Diameter with a sample size (n) of 39. (**B**,**C**) The red line represents a Gaussian distribution fit curve and blue dashed line represents the mean length and diameter respectively.

The CdSe/ZnS QDs exhibited an ellipsoidal shape (Figure 2A) when visualized by TEM. The average length of our QDs was 8.87 ± 1.38 nm with an average diameter of 4.57 ± 0.52 nm (Figure 2B,C). The measured diameter is consistent with the size reported by the manufacturer. However, some particle agglomeration was observed in the TEM images (Figure 2A). Therefore, to minimize agglomeration, the QDs were thoroughly sonicated before performing further experiments.

**Figure 3:**
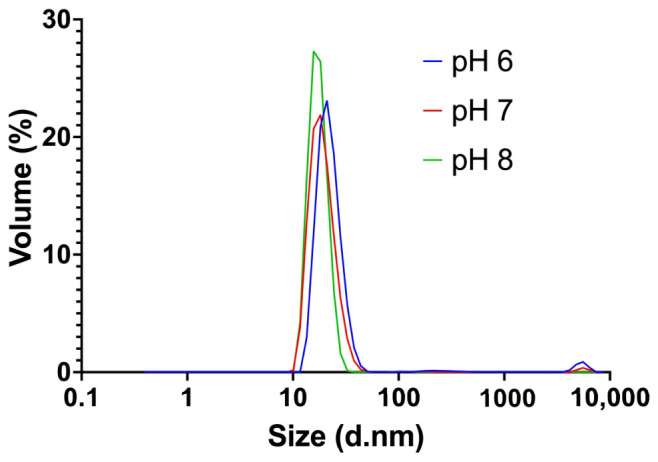
Volume distribution representing the likely makeup of QDs using Dynamic Light Scattering (DLS). Samples were run in triplicate for each pH.

After sonication, volume-based particle size distribution revealed that the average particle size of the QDs was broadly similar across all the pH conditions tested. The mean hydrodynamic diameters were 22.68 ± 6.616 nm, 20.07 ± 5.810 nm, and 18.35 ± 4.407 nm for pH 6, 7, and 8, respectively (Figure 3). Although there was a slight decrease in the average particle size as pH increased, the overlap in size across the different pHs suggests that the QDs’ size remains comparable. The single dominant peak further indicates that the majority of the QD samples are well dispersed. Additionally, QDs’ size in DLS is larger compared to the TEM analysis, as DLS measures the hydrodynamic size, which includes the hydration layers attached to the QDs, making it appear larger, so the true QD size is likely closer to our TEM analysis.

### 2.2. Fluorometric Assessment of α-Actinin–QDs Binding

**Figure 4:**
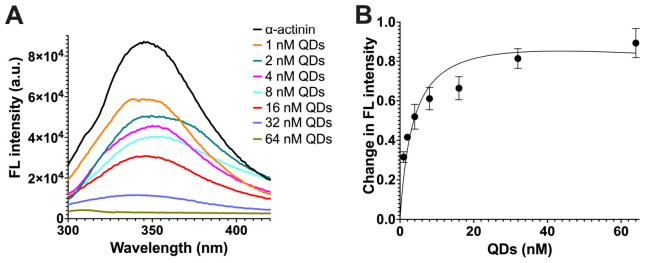
Quenching of intrinsic fluorescence of α-actinin in the presence of CdSe/ZnS QDs. (**A**) The intrinsic fluorescence of α-actinin when treated with different concentrations of QDs. This experiment represents one of the triplicated experiments. (**B**) The K_d_ estimation between α-actinin and QDs using the one site–total binding analysis. The graph represents the mean and the standard error in triplicate.

α-actinin emits an intrinsic fluorescence due to the presence of the amino acid tryptophan in their structure [15]. An alteration in the fluorescence intensity would be indicative of interactions between the fluorophore (α-actinin) and a quencher molecule (QDs) [16,17]. Therefore, we carried out a quenching assay that showed that the intrinsic fluorescence of α-actinin decreased as they were incubated with QDs. We found that the higher the concentration of QDs, the more quenching of the intrinsic fluorescence at 340 nm (Figure 4A). For example, 32 nM QDs caused 86.9% quenching compared to only 32.4% quenching at 1 nM QD treatment (Figure 4B). Using the one site–total binding analysis, the disassociation constant, K_d_, was estimated to be 3.70 ± 0.55 nM, indicating high binding affinity between QDs and α-actinin.

### 2.3. Gel Assessment of α-Actinin–QDs Binding

**Figure 5:**
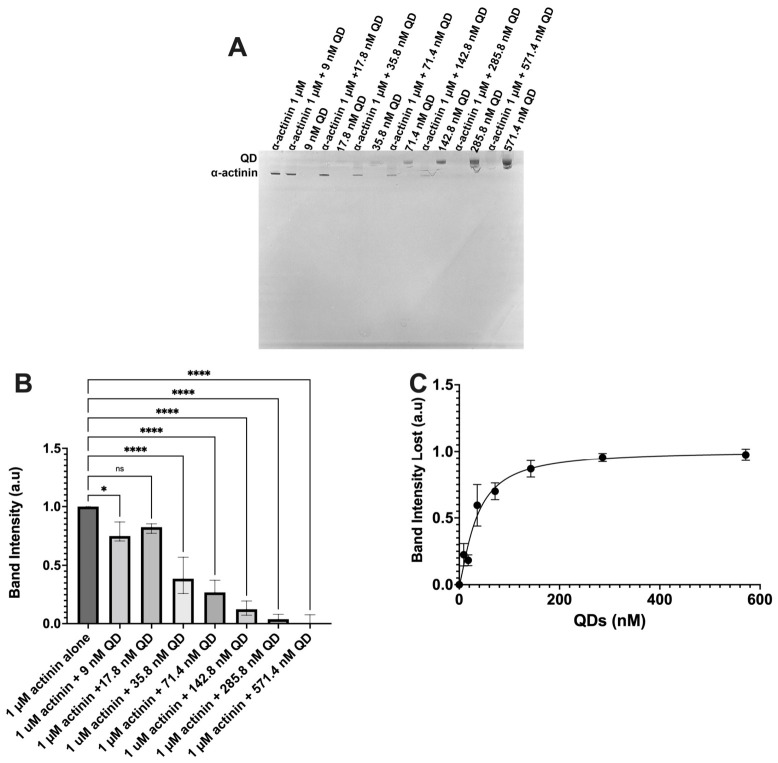
Native PAGE of α-actinin in the presence of CdSe/ZnS QDs. (**A**) Representative Native gel of 1 µM α-actinin incubated with increasing concentrations of QDs (9–571.4 nM). (**B**) Quantification of α-actinin band intensity, where n = 3. (**C**) Graph of α-actinin band intensity decrease as QD concentration increases. Data were fitted using the Hill equation to model the apparent K_d_. The graph represents the mean and the standard error in triplicate. * *p* < 0.05, **** *p* < 0.0001.

In addition to intrinsic fluorescence quenching, a native Polyacrylamide Gel Electrophoresis (PAGE) was used to further investigate the interactions between α-actinin and QDs. Here, in the control sample containing α-actinin only, a distinct band was observed. Similar bands were observed in samples where α-actinin was mixed with a lower concentration of QDs (9–35.8 nM). However, as QD concentration increased (71.4–571.4 nM) in the samples, the intensity of the α-actinin band decreased subsequently (Figure 5A,B). The lowered intensity of the α-actinin band at higher QD concentrations is consistent with formation of α-actinin–QD complexes and indicative of an increasing amount of α-actinin being associated with QDs. Furthermore, using the Hill equation [18], the apparent K_d_ was determined to be 34.27 ± 4.998 nM (Figure 5C) with a 95% CI [23.88, 44.67]. Likewise, the Hill coefficient (h value) was 1.316 ± 0.2193 with a 95% CI [0.86, 1.77], demonstrating positive cooperativity.

### 2.4. DLS Assessment of α-Actinin–QDs Binding

**Figure 6:**
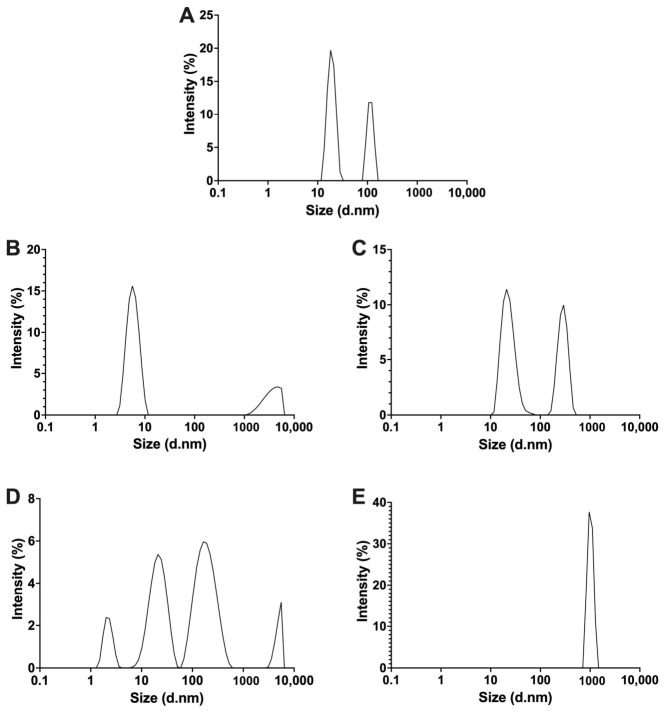
DLS intensity distribution of (**A**) CdSe/ZnS QDs, (**B**) BSA, (**C**) BSA–QDs, (**D**) α-actinin, (**E**) α-actinin–QDs.

DLS intensity distribution analysis was used to evaluate the hydrodynamic size of QDs in the absence and presence of α-actinin. QDs alone exhibited two peaks at 19.10 ± 3.364 nm (66% intensity) and 114.6 ± 15.61 nm (34% intensity) (Figure 6A), indicating a majority of QDs having a small size with some aggregates. Notably, the secondary peak at 114.6 ± 15.61 nm was not observed in the volume distribution graph (Figure 3). This discrepancy arises because in intensity-based measurements, larger particles scatter more light compared to smaller particles. Therefore, even a smaller population of aggregates can result in an intense signal. For BSA alone, a major peak (77% intensity) was observed at 5.881 ± 1.558 nm (Figure 6B). Upon QD treatment, the BSA–QDs sample exhibited peaks at 23.53 ± 7.756 nm and 289.0 ± 62.25 nm (Figure 6C). This suggests an increase in particle size relative to both QDs and BSA. α-actinin alone showed a heterogeneous size distribution with major peaks at 249.4 nm ± 113.2 nm (45.2% intensity) and 29.27 ± 12.13 nm (36.6% intensity) and a minor peak at 6.430 nm (9.5% intensity) (Figure 6D). On the contrary, the α-actinin–QDs mixture exhibited a single dominant peak at 980.1 ± 151.5 nm (Figure 6E). The disappearance of smaller particle sizes and presence of a large hydrodynamic structure further suggest complex formation following addition of α-actinin to QDs.

### 2.5. Gel Assessment of Actin–α-Actinin–QDs Binding via Spin-Down Assay

As mentioned earlier, α-actinin is a well-known actin crosslinking protein that connects adjacent actin filaments to form parallel or anti-parallel actin bundles [14,15]. Given our previous study showed that a high concentration of QDs can alter F-actin dynamics and promote actin bundling [11], we set out to determine whether QDs affect α-actinin-mediated actin bundling. For this, we performed a low-spin (7000 rpm) spin-down assay, followed by Sodium Dodecyl Sulfate–Polyacrylamide Gel Electrophoresis (SDS-PAGE) analysis. In the low-speed spin-down assay, bundled actin filaments settle down into the pellet during centrifugation, whereas the unbundled actin remains largely in the supernatant [19,20].

**Figure 7:**
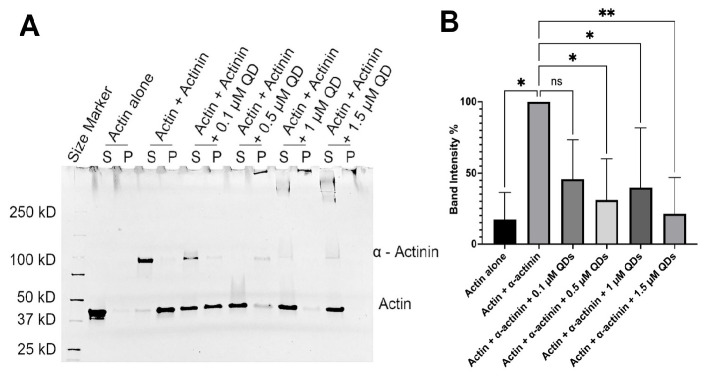
Spin-down assay to assess α-actinin-mediated actin bundling in the presence of QDs. The assay was performed at low-speed centrifugation (7000 rpm). (**A**) SDS gel showing actin bundling assessment. The supernatant (S) and pellet (P) of each sample are located next to each other. (**B**) Quantification of the actin band intensity from the pellet of each sample, where n = 4. Statistical analysis was carried out using Dunnett’s multiple comparisons test, * *p* < 0.05, ** *p* < 0.01.

Our result showed that the alpha-actinin-mediated actin bundling decreased as increasing concentrations of QDs were introduced (Figure 7A,B). In the control sample containing actin and α-actinin without QDs, a thick actin band was observed in the corresponding pellet lane, demonstrating the efficient bundling of actin filaments. At 0.1 µM QD treatment, a prominent actin band was still observed, whereas the higher QD concentrations resulted in noticeably weaker actin bands (Figure 7A,B). This suggests that substantial actin bundling occurs at lower QD concentrations, while higher concentrations strongly reduce the α-actinin-mediated actin bundling.

### 2.6. QDs Mediated Depolymerization of Actin Filaments in the Presence of α-Actinin

α-actinin stabilizes actin filaments and slows down their depolymerization [21], whereas QDs have been shown to cause rapid depolymerization [11]. Thus, we investigated the actin depolymerization kinetics in the presence of both QDs and α-actinin by measuring the decrease in normalized fluorescence intensity over time. In this fluorescence-based assay, higher fluorescence is indicative of the presence of more polymerized F-actin and decreasing fluorescence corresponds to actin filament depolymerization.

**Figure 8:**
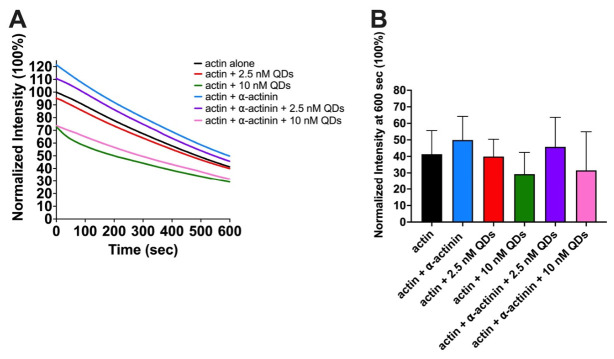
Assessment of F-actin depolymerization. (**A**) F-actin filaments were incubated with differing concentrations of QDs to measure the F-actin depolymerization in the absence or presence of alpha-actinin. This graph represents a triplicate experiment. (**B**) Quantification of actin’s fluorescent intensity at 600 s. This graph represents a triplicate experiment.

Here, the fluorescent intensity of actin alone decreases from 100% at 0 s to 41% after 600 s (Figure 8A,B), which represents the intrinsic depolymerization rate of F-actin. The addition of 2.5 nM QDs resulted in a similar fluorescent intensity of around 39%, whereas upon 10 nM QD treatment, depolymerization accelerated, resulting in only 29% intensity after 600 s. As expected, the presence of α-actinin helped stabilize the actin filaments by maintaining relatively higher fluorescence throughout and retaining 50% intensity at 600 s. However, when QDs were introduced in the presence of α-actinin, its stabilizing effect was reduced in a dose-dependent manner: actin in the presence of α-actinin and 2.5 nM QDs retained 45% intensity at 600 s compared to only 31% upon 10 nM QD treatment (Figure 8A,B).

### 2.7. Computational Model Construction of QDs–α-Actinin Complexes

To complement the biochemical measurements, we built atomistic CdSe/ZnS-derived QD interaction models with α-actinin using a scripted Python workflow (ASE, RDKit, NumPy). The ZnS shell was generated from a zinc blende ZnS lattice (lattice constant 5.41 Å) and trimmed to a spherical nanoparticle. Consequently, surface ligand placement was performed using DHLA as the ligand to generate coated QDs with controlled spatial distribution of anchoring sites. Two coated QD chemistries were then used for the final comparison: a deprotonated coated QD with a carboxylate-enriched surface (COO^−^) and a protonated coated QD with a carboxylic acid surface (COOH). This design allowed us to screen the effect of protonation and deprotonation state on protein interaction while preserving the same core/shell geometry and patch-screening logic.

**Figure 9:**
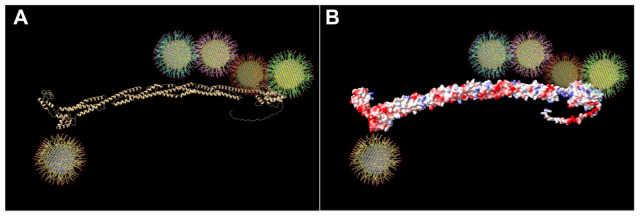
Electrostatic patch placement of coated QDs on α-actinin shown via (**A**) Ribbon diagram and (**B**) Electrostatic patch model. The structure of α-actinin was generated using AlphaFold (UniProt ID: A0A5F9DPI3, ~30–40 nm length) [22], with the electrostatic patch model used to identify potential QD (~6 nm diameter) interaction regions. The surface coloring represents electrostatic charge distribution, where red indicates negatively charged regions, blue indicates positively charged regions and white indicates the neutral regions. While the figures indicate all the potential binding sites collectively, molecular dynamics simulation was performed with a single QD interacting with α-actinin at one patch at a time.

Potential interaction regions on α-actinin were selected algorithmically using local net-charge scoring, in which formal residue charges (ASP/GLU = −1, LYS/ARG/HIS = +1, others = 0) were summed within a 15 Å radius around each Cα position. Five patches (one negative, three positive, one neutral) were then selected with a minimum separation constraint between patch centers (Figure 9A,B). Adaptive Poisson–Boltzmann Solver (APBS) electrostatic surfaces visualized in ChimeraX were used to illustrate these selected regions rather than to compute patch scores. For each patch, both QD chemistries (protonated and deprotonated) were placed with a uniform initial QD–protein minimum separation target, producing 10 systems in total (2 chemistries in each 5 patches).

### 2.8. Molecular Dynamics and 5 Nanoseconds (ns) Trajectory Analysis

**Figure 10:**
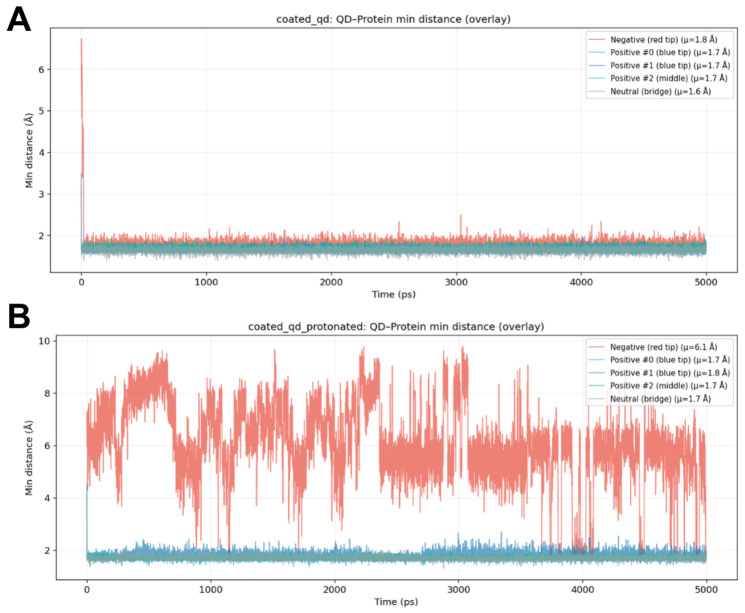

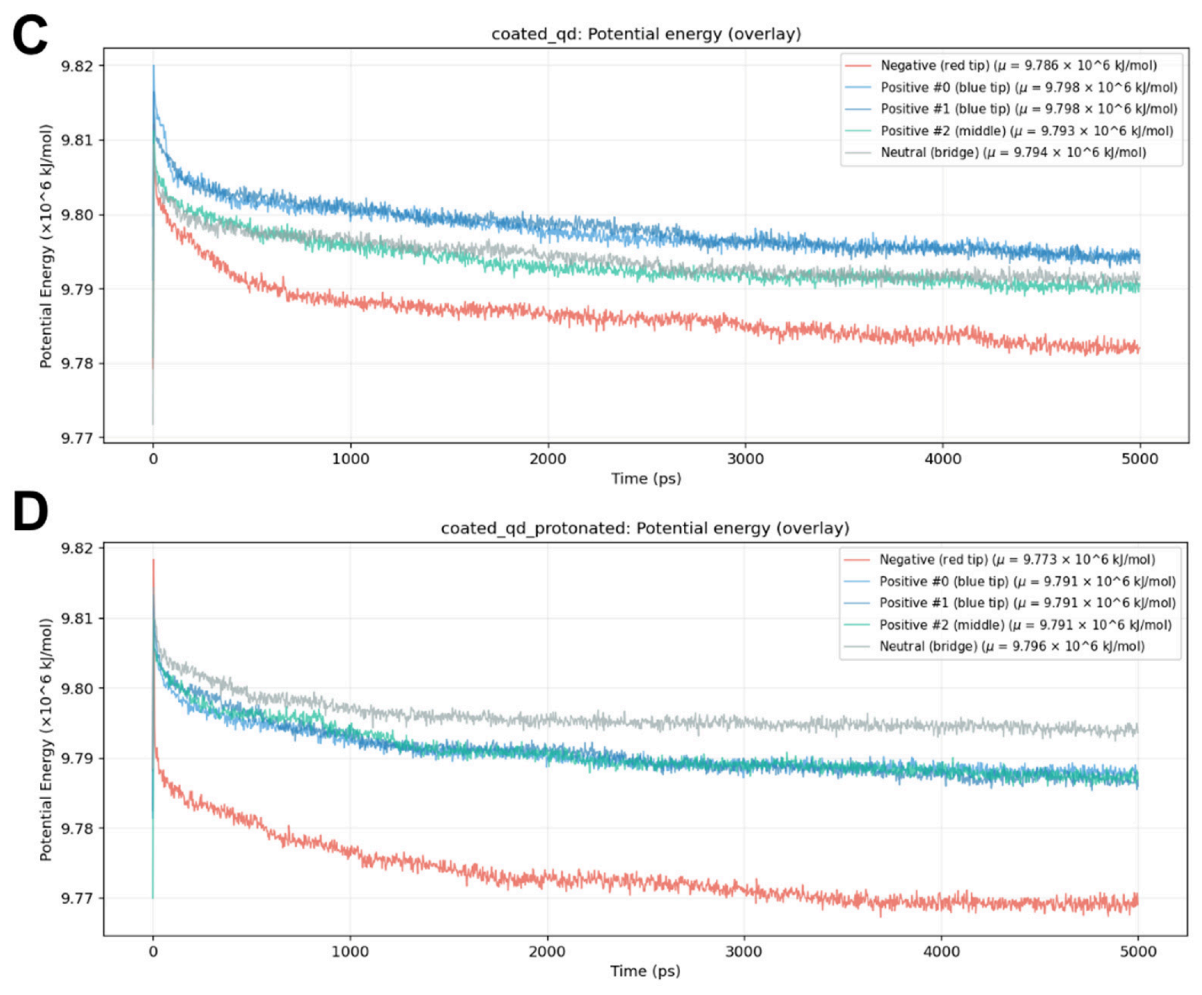
5 ns interaction stability: minimum distance and potential energy. (**A**) Mean QD–protein minimum distance vs. time for deprotonated QD across 5 patches. (**B**) Mean QD–protein minimum distance vs. time for protonated QD across 5 patches. (**C**) Potential energy vs. time for deprotonated coated QD patch systems. (**D**) Potential energy vs. time for protonated coated QD patch systems.

All 10 systems completed 5 ns molecular dynamics simulations at 310.15 K. Across both coated-QD chemistries, trajectories generally maintained near-contact QD–protein configurations, with patch fluctuations in minimum separation (Figure 10A,B). Mean QD–protein minimum distance profiles showed that most patch trajectories remained in a stable near-contact regime, with one protonated negative-patch trajectory displaying a transient long-distance excursion before returning to near contact by the end of the run (Figure 10B). In parallel, the potential energy remained stable across the simulation windows, supporting dynamical consistency of the runs (Figure 10C,D).

Overall, the 5 ns molecular dynamics simulations revealed that QDs form and maintain persistent near-contact configurations with α-actinin across all the tested electrostatic patches regardless of their protonation state. This indicates the kinetic tendency toward contact maintenance of QD–α-actinin interactions under physiological conditions and any variation that occurs is most likely due to the surface chemistry and the local protein environment.

**Figure 11:**
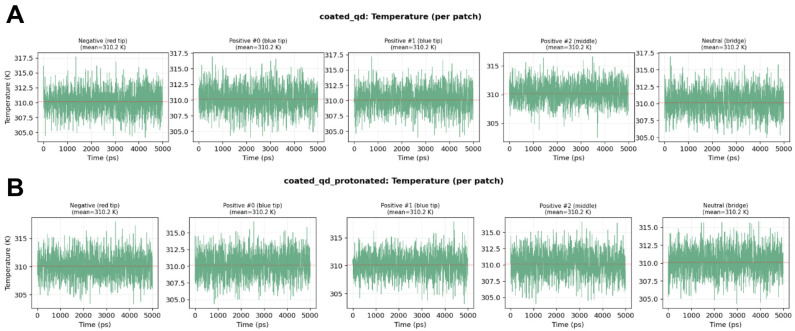
Temperature stability during 5 ns simulations. (**A**) Representative temperature trace (deprotonated coated QD). (**B**) Representative temperature trace (protonated coated QD). The red dashed line in each subplot indicates the mean temperature of that corresponding simulation trajectory, with the mean value reported in the subplot title. The mean temperature remained close to the target simulation temperature of 310.2 K.

Temperature control was highly stable across all simulations (clustered near 310 K) (Figure 11A,B), indicating that the differences observed in distance behavior were associated with interaction and placement effects rather than thermostat instability.

To identify local binding environments, residues were ranked by minimum residue-to-QD-center distance over the analyzed trajectories and summarized as the top 10 closest residues for each chemistry (Table 1). The closest residue set was enriched in charged and polar residues, consistent with electrostatic steering of local interface formation. Figure 7 reports atom–atom minimum QD–protein distances, whereas Table 1 reports residue-to-QD-center distances; these metrics are complementary and should not be compared numerically as the same quantity.

**Figure 12:**
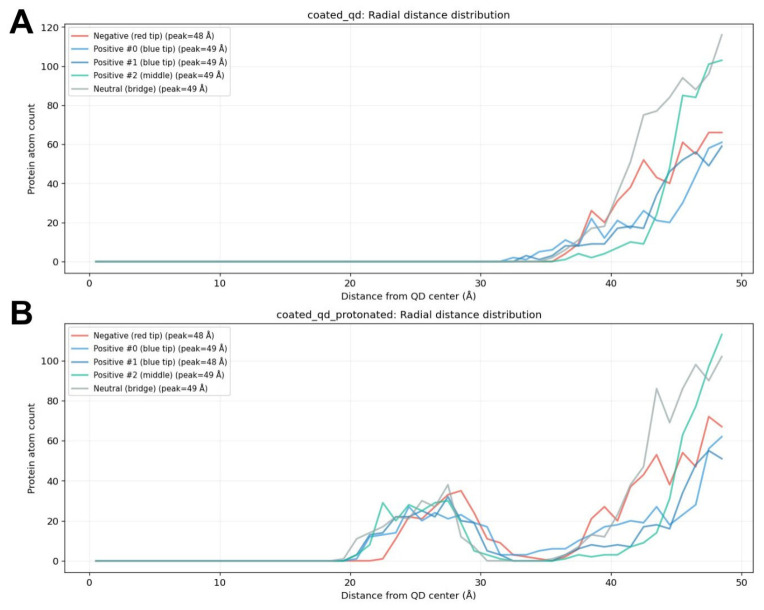
Radial distance distribution of protein atoms around QD center (5 ns). (**A**) Deprotonated coated QD systems (five patches). (**B**) Protonated coated QD systems (five patches).

Radial distance distributions further showed chemistry- and patch-dependent structuring of protein atoms around the QD center (Figure 12A,B), supporting the same interaction trends observed in the minimum distance analysis.

Taken together, the 5 ns simulations indicate that coated QD protonation state and local protein patch chemistry jointly modulate QD–α-actinin contact persistence and residue-level engagement.

## 3. Discussion

Here, to our knowledge, this is the first study that reveals that in addition to binding to actin [10,11], CdSe/ZnS QDs interact with actin crosslinking protein, α-actinin, and consequently affect actin filament stability. By integrating biochemical experiments and physics-based computational modeling, we provide evidence that QDs disrupt the actin cytoskeleton through both direct interactions with actin and interfering with the intrinsic activity of α-actinin in stabilizing F-actin filaments.

The QDs used in this study have a net negative surface charge of −30.4 mV (Figure 1C), suggesting their tendency to have electrostatic interactions with positively charged regions of their binding protein partners. Our computational modeling supports this idea, as the predicted binding configurations demonstrated reduced binding distances between the QD carboxylate groups (COO^−^) and positively charged surfaces on α-actinin compared to the negatively charged surface. This suggests that electrostatic forces are a primary force for QDs–α-actinin interaction. This is further supported by the molecular dynamics simulations where positively charged residues like Arg and Lys contribute strongly to electrostatic protein–ligand interactions and are among the closest residues to the QDs’ surface. That said, we would like to note that the persistence of near-contact distances throughout the simulations suggests that QDs can have stable interactions with α-actinin under physiological conditions. We acknowledge that the 5 ns simulation windows demonstrate kinetic persistence of near-contact configurations rather than fully converged thermodynamic binding affinity; longer timescale simulations or free energy calculations would be required to quantify absolute binding strength. Additionally, our residue proximity to QDs via analyzed trajectories (Table 1) shows that QDs interact with the actin-binding domain (ABD) of α-actinin. The ABD of α-actinin consists of tandem calponin homology (CH) domains, CH1 and CH2, which tentatively range between residues 26 and 250 [23]. We speculate that such stable interactions between QDs and α-actinin may cause steric hindrance and interfere with the actin-binding domains of α-actinin, causing QDs to impair α-actinin’s crosslinking function. As such, additional studies and experimental verifications are required to determine whether actin-binding or crosslinking activity is affected.

Experimentally, fluorescence quenching and native PAGE assays revealed a dissociation constant (K_d_) of 3.7 nM and 34.3 nM, respectively, for QD–α-actinin interaction. This binding affinity is substantially higher than the K_d_ of 400 nM between QDs and globular actin (G-actin) reported by Le et al., which was determined by a native PAGE [10]. There are several factors that may account for this difference. α-actinin (30–40 nm in length, 3–4 nm wide) is a significantly larger protein compared to G-actin (3.5 nm thick, 5.5 nm height and width) [24,25], so it provides a larger interaction surface for QDs binding. Additionally, the difference in methodological approach may also contribute to the discrepancy in K_d_ values obtained in the present study, as gel-based assays may underestimate the binding affinity compared to fluorescence-based quenching, which is very sensitive to environmental changes around the tryptophan residues.

The DLS intensity distribution (Figure 6) suggested that QDs interact differently with BSA and α-actinin. Incubation of QDs with BSA resulted in the formation of larger particles while retaining smaller-sized particles as well. In contrast, incubation of QDs with α-actinin resulted in the disappearance of smaller-sized particles and presented only one distinct large particle population. This difference could be related to the protein and its structural properties. Having multiple interaction domains may enable multiple QDs to bind to α-actinin simultaneously, resulting in extensive particle clustering. This raises the possibility that interactions between QDs and cytoskeletal proteins could promote nanoparticle aggregation within biological environments. Further studies need to be carried out to assess its biological implications and contribution to nanotoxicity.

Furthermore, QD–α-actinin binding had functional consequences on actin organization. The spin-down assay demonstrated that actin bundling was inhibited even in the presence of α-actinin after QD treatment, suggesting impairment in crosslinking activity. Actin bundling is essential for organizing actin into higher-order structures for key processes such as cell division, migration, invasion, and bulk transport [14]. Therefore, the disruption of the actin bundling process implies that QDs can compromise higher-order actin architecture. The effects of QDs on the cytoskeletal proteins, actin and α-actinin, were further demonstrated in the depolymerization assay. As reported in our previous study as well [11], the presence of QDs caused more significant depolymerization. Here, α-actinin showcased its stabilizing effect by slowing down actin depolymerization, which is consistent with previous reports [26]. Likewise, consistent with our findings, other actin bundling proteins such as the *Dictyostelium* 30 kDa actin-bundling protein, plastin and fascin, have been reported to stabilize F-actin by inhibiting depolymerization [27,28,29]. Consequently, a clear trend was observed, where in the presence of QDs, this stabilizing effect was weakened, suggesting that QD binding interferes with α-actinin’s regulatory function. The variability observed in the depolymerization data is due to how relative the fluorescent intensity is during each independent experimental run, which is why normalization was carried out to enable comparison of depolymerization kinetics.

As such, the biological implications of our findings are significant. As mentioned earlier, the actin cytoskeleton and its organization is fundamental to processes such as cell migration, adhesion, division, and muscle contraction. Perturbations in actin bundling and filament stability could contribute to compromised structural integrity and impaired motility. Furthermore, from a nanotoxicology perspective, our results suggest that QD-induced actin cytoskeletal dysfunction may arise not only from direct interaction but also from interference with regulatory protein networks. This expands on the current understanding that QDs influence intracellular systems and, in particular, the actin cytoskeleton.

Depending on the biological context, these perturbations in actin dynamics may be either detrimental or beneficial. As such, identifying the specific residues involved in protein–QD interactions may enable the rational design of surface functionalized QDs that either enhance or minimize such interactions by selectively targeting particular protein domains. This improves precision drug delivery while reducing unintentional cytoskeletal toxicity.

Therefore, future research should focus on expanding these findings into more complex biological systems. Studying QDs and actin/α-actinin interactions in muscle cells would provide physiological consequences in a cellular context. This might also demonstrate the potential of QDs in regenerative medicine. Additionally, transitioning from heavy metal-based QDs to carbon-based quantum dots will help minimize toxicity while preserving functional tunability. From a computational perspective, expanding simulations to include multiple QDs within protein-rich environments could better approximate intracellular conditions and provide insight into competitive binding environment.

## 4. Materials and Methods

### 4.1. QDs’ Characterization

We obtained visible water-soluble CdSe/ZnS QDs coated with carboxylic acid terminal end groups from NanoOptical Materials Inc. (Carson, CA, USA). To ensure nanoparticle integrity and consistency with expected physical/chemical properties, characterization studies using spectroscopy, zeta potential, and TEM were carried out.

For fluorometer emission, we diluted the stock concentration of QDs (11 µM) to 100 nM using deionized water. The excitation and emission wavelength were adjusted to 280 nm and 350–700 nm respectively, and the emission spectra were measured using a PTI spectrofluorometer (PTI Photon Technology International, Birmingham, NJ, USA). The excitation of QDs at 280 nm is very efficient with high fluorescence intensity, which results in better image quality [30], whereas the emission wavelength range of 350–700 nm allows us to fully capture the fluorescence spectrum of CdSe/ZnS QDs and detect any potential overlap with α-actinin fluorescence.

For zeta potential, QDs were similarly diluted to 100 nM and the electric potential of QDs was measured using a Zetasizer Nano ZS90 (Malvern Panalytical, Westborough, MA, USA).

For UV-Vis, a fixed concentration of 50 nM of QDs was used in Millipore water (pH 8.0) to create our samples. The absorbance of the samples was scanned from 350 to 750 nm with a UV-2101PC UV–Vis spectrophotometer (Shimadzu, Columbia, MD, USA). The data was then graphed using GraphPad Prism 9.

For TEM, the data was obtained by sending our sample of QDs in a pH 7 phosphate buffer to the University of Missouri, and they used a JEM-1400 (JEOL USA, Peabody, MA, USA).

For DLS, QDs with a concentration of 100 nM were prepared in a variety of phosphate buffers, with pH values of 6, 7, and 8. We measured the size of the quantum dots using a refractive index of 2.80 and an absorption value of 1.00 using the Zetasizer. The Volume Distribution graph was used to estimate the hydrodynamic size of the QDs. The data was then graphed using GraphPad Prism 9.

### 4.2. Actin Preparation

We homogenized 1 mg of pyrene-labeled rabbit skeletal muscle actin (Cytoskeleton Inc., Denver, CO, USA) in 50 µL cold sterile deionized water to prepare a stock concentration of 20 mg/mL (465.12 µM) and kept it on ice. For the fluorometric assay, we further diluted the actin in general actin (G) buffer (5 mM Tris-HCl, pH 8.0, 0.2 mM CaCl_2_, 0.2 mM ATP) to a working concentration of 4 µM. Next, the diluted actin was introduced with 1X actin polymerization (AP) buffer (500 mM KCl, 20 mM MgCl_2_, 50 mM Guanine Carbonate, 10 mM ATP, 100 mM Tris) and incubated for 1 h at room temperature. The actin was then ready for experimental use.

For the spin-down assays, we diluted the actin to a working concentration of 5 µM and incubated it on ice for 30 min. Next, the diluted actin was introduced with 1X AP buffer and incubated for 1 h at room temperature before it was ready for experimental use.

### 4.3. α-Actinin Preparation

For all the assays, we homogenized 50 µg α-actinin (Cytoskeleton Inc., Denver, CO, USA) in 50 µL cold sterile deionized water to prepare a stock concentration of 1 mg/mL (10 µM) and kept it on ice. In fluorometric assays, the α-actinin was further diluted in the F-actin mixture to a working concentration of 0.112 µM, while for the spin-down assays, it was diluted to a working concentration of 0.5 µM.

### 4.4. Evaluation of Protein and QD Binding via Dynamic Light Scattering (DLS)

We completed 5 runs in total. The first run was 100 nM QD alone, second was 1 µM BSA alone, third was 1 µM BSA + QD, fourth was 1 µM α-actinin alone, and fifth was 1 µM α-actinin and 100 nm QDs. These runs were all done at room temperature in a pH 8 phosphate buffer. We used a refractive index of 2.80 and an absorption value of 1.00. The machine that was used for DLS was the Zetasizer. We used the intensity distribution graph to represent this data set.

### 4.5. Native Gel Electrophoresis

α-Actinin with a fixed concentration of 2 µM and variable QD concentrations of 9 nM, 17.8 nM, 35.8 nM, 71.4 nM, 142.8 nM, 285.8 nM, and 571.4 nM were mixed. Samples of varying concentrations of QDs and fixed concentrations of α-actinin were also prepared as controls. G-buffer (5 mM Tris HCL, pH 8, 0.2 mM CaCl_2_, and 0.2 mM ATP) was added to make all samples’ total volume 10 µL. The samples were incubated for 3 h at room temperature in the dark. While the samples were incubated, a Mini-PROTEAN precast polyacrylamide gel (Bio-Rad, Hercules, CA, USA) was pre-run in a native gel buffer (25 mM Tris-HCl, pH 8, 194 mM glycine, 0.5 mM CaCl_2_, 0.2 mM DTT, and 0.2 mM ATP) for 1 h at 70 V on ice. The incubated samples were then pipetted into their respective lanes, and the gel was run at 190 V for 50 min. The gel was then stained using a Coomassie brilliant blue R-250 dye for 30 min. After dumping out the dyeing solution, the gels were destained overnight, and images of the gel were taken. For the Native gel assessment, band intensity was quantified using relative densiometric analysis with Image J 1.35t. The values were then converted to relative band intensity lost using the equation $1-\frac{B}{B_{0}}$, where *B* = band intensity of α-actinin + QDs, and $B_{0}$ = band intensity of α-actinin alone.

### 4.6. Assessment of Actin and α-Actinin Quenching by Fluorometer-Based Assay

The F-actin and α-actinin stock was prepared as mentioned in Section 4.2 and Section 4.3 respectively. We added different concentrations of QDs right before experimental use.

We measured the emission spectra of actinin–QD binding (Section 2.2) by adjusting the excitation and emission wavelength to 280 nm and 300–450 nm respectively. The excitation wavelength of 280 nm was chosen because the tryptophan residues in α-actinin absorb strongly at this wavelength, while the emission wavelength range of 300–450 nm allows us to fully capture the fluorescence spectrum of α-actinin [17]. For the dissociation constant (K_d_), the fluorescent intensity of α-actinin was measured at 345 nm in the absence and presence of varying concentrations of QDs. The fluorescence values were then normalized such that the fluorescent intensity of α-actinin alone was set to a value of 1. The relative change was then calculated as $1-\frac{F}{Fo}$, where *F* = fluorescent intensity of α-actinin + QDs, and *Fo* = initial fluorescent intensity of α-actinin.

For the emission spectra of pyrene actin–α-actinin–QDs binding (Section 2.4), we measured the fluorescent signal over time (600 s) with the standard excitation and emission wavelength of 365 nm and 407 nm respectively.

### 4.7. Spin-Down Assay Followed by SDS-PAGE

A fixed concentration of 5 µM actin and 0.5 µM α-actinin was prepared as mentioned in Section 4.2 and Section 4.3 respectively. We created the testing samples by mixing the actin and actinin with F-buffer (G buffer + AP buffer), Tris-HCl, pH 6.5, and different concentrations of QDs (0.1, 0.5, 1, and 1.5 µM) in individual Eppendorf tubes (e-tubes). We then incubated the e-tubes away from light for 30 min at room temperature and ultracentrifuged them at 14,000× *g* (7000 rpm) for 1 h at 24 C. After centrifugation, we removed the supernatant and placed it in separate e-tubes on ice. The pellets were also resuspended separately in 40 µL F-buffer by pipetting up and down for 3 min and placed on ice. Next, we mixed 10 µL of 6X Laemmli SDS buffer into each e-tube before heating them at 95 C for 4 min. Finally, we used a mini-protean TGX precast gel (Bio-Rad, Hercules, CA, USA) to load and run the samples at 180 V for 45 min. The gels were then stained with Coomassie Blue, and images were taken with a gel scanner. The band intensity was quantified using relative densiometric analysis with Image J.

### 4.8. Data Analysis

All the experiments were performed in triplicate. QD characterization, fluorometric assessments, and all quantitative analyses were processed and graphed using GraphPad Prism 9. TEM images were quantified using Image J.

The data in the histograms (Figure 2B,C) are presented as average ± standard deviation. The binding affinity (K_d_) for the fluorometric analysis was determined by fitting the quenching data to a one site–total binding isotherm with constraints Bmax = 1 and background = 0 in Prism. For the native gel analysis, the binding affinity (K_d_) was determined by fitting the relative band intensity lost values into the standard Hill equation, also using Prism. For the SDS gel assessment, band intensities were quantified using Image J and then normalized to the positive control (actin + α-actinin), which had 100% band intensity. Statistical significance was assessed using one-way ANOVA in Prism.

### 4.9. QD Model Generation

QD structures were generated using Python 3.11.14 with ASE 3.26.0, RDKit 2025.09.2, and NumPy 2.3.4. A spherical ZnS core was constructed from the zinc blende ZnS crystal structure (lattice constant a = 5.41 Å) and trimmed to produce a nanoparticle core geometry. Ligand placement and coating construction were automated with recorded metadata for atom mapping, ligand anchoring, and topology reproducibility.

For the final simulation campaign, coated deprotonated and coated protonated QD models were prepared to compare surface protonation effects while maintaining identical core geometry.

ZnS bonded and nonbonded interaction terms were implemented using literature-derived ZnS interaction parameters (Table 2) previously developed for ZnS molecular dynamics simulations and later applied in biomolecular adsorption studies [31,32]. These parameters include harmonic bond and angle terms, partial atomic charges, and Lennard–Jones interactions describing Zn-S interactions within the ZnS lattice.

### 4.10. Molecular Dynamics System Preparation and Simulation Protocol

α-actinin patch targets were selected from electrostatic patch mapping and used to place each QD model at five defined regions (negative_0, positive_0, positive_1, positive_2, neutral_0). Initial complex geometries were adjusted to satisfy a consistent minimum QD–protein placement criterion.

Protein and QD topology components were assembled into system directories using the GROMACS 2025.4 simulation package [33]. The α-actinin protein was described using the AMBER99SB-ILDN force field [34], while QD topology files were generated from the custom ZnS parameter workflow described above.

Simulation systems were placed in cubic periodic boxes without explicit solvation or ion addition. Production MD simulations used a vacuum-style setup with pcoupl = no, coulombtype = Cut-off, rcoulomb = 1.4 nm, vdwtype = Cut-off, rvdw = 1.4 nm, and pbc = xyz. No separate solvation or ion-neutralization step was performed.

Energy minimization was performed using the steepest descent algorithm before molecular dynamics simulations. All systems were simulated for 5 ns at 310.15 K using identical parameters to allow direct comparison between coated-QD chemistries and placement patches.

Production MD simulations used a timestep of 0.001 ps with the Verlet cutoff scheme. Electrostatic interactions were treated using a cutoff distance of 1.4 nm, and Lennard–Jones interactions were truncated at 1.4 nm. Temperature was controlled using the velocity-rescale thermostat with a reference temperature of 310.15 K.

Periodic boundary conditions were applied in all spatial directions. Identical simulation parameters were applied across all ten systems to ensure consistent comparison of QD–protein interaction behavior.

## Figures and Tables

**Table 1:** Top 10 closest α-actinin residues to coated QDs in 5 ns simulations, ranked by minimum residue-to-QD-center distance.

Rank	Residue	Patch Region	Charge	Min. Distance to QD Center (nm)	Chemistry
1	ARG89	Positive #0	+1	3.27	Deprotonated
2	LYS202	Positive #1	+1	3.32	Deprotonated
3	TYR417	Neutral bridge	0	3.56	Deprotonated
4	ARG299	Positive #2	+1	3.65	Deprotonated
5	ILE782	Negative	0	3.63	Deprotonated
6	ARG89	Positive #0	+1	3.41	Protonated
7	LYS91	Positive #0	+1	3.47	Protonated
8	TYR417	Neutral bridge	0	3.57	Protonated
9	ARG299	Positive #2	+1	3.68	Protonated
10	ILE782	Negative	0	3.66	Protonated

**Table 2:** ZnS interaction parameters used in this study (adapted from prior ZnS parameterizations [31,32]).

Bond Parameters:			
**Bond**	**k (kJ mol^−1^ nm^−2^)**		**r_o_ (nm)**
Zn–S	92,000		0.16
Angle Parameters:			
**Angle**	**k (kJ mol^−1^ rad^−2^)**		**θ_o_ (degrees)**
S–Zn–S	274.022		109.47°
Zn–S–Zn	274.022		109.47°
Atomic Charges:			
**Atom**		**Charge (e)**	
Zn		+2.0	
S		−2.0	
Lennard–Jones Parameters:			
**Atom**	**σ (nm)**		**ε (kJ mol** **^−^** **^1^** **)**
Zn	0.3816		0.022
S	0.4270		1.087

## Data Availability

Data are available upon request.

## Abbreviations

The following abbreviations are used in this manuscript:

QDs	Quantum Dots
ABD	Actin Binding Domain
CH	Calponin Homology
DHLA	Dihydrolipoic Acid
TEM	Transmission Electron Microscopy
SDS PAGE	Sodium Dodecyl Sulfate Polyacrylamide Gel Electrophoresis
APBS	Adaptive Poisson Boltzmann Solver
MD	Molecular Dynamics
F actin	Filamentous Actin
G actin	Globular Actin
DLS	Dynamic Light Scattering

## References

[1] Adepu S., Ramakrishna S. (2021). Controlled Drug Delivery Systems: Current Status and Future Directions. Molecules.

[2] Gupta D., Gupta S.V., Yang N. (2022). Understanding the Routes of Administration. Handbook of Space Pharmaceuticals.

[3] Wen H., Jung H., Li X. (2015). Drug Delivery Approaches in Addressing Clinical Pharmacology-Related Issues: Opportunities and Challenges. AAPS J..

[4] Ezike T.C., Okpala U.S., Onoja U.L., Nwike C.P., Ezeako E.C., Okpara O.J., Okoroafor C.C., Eze S.C., Kalu O.L., Odoh E.C. (2023). Advances in drug delivery systems, challenges and future directions. Heliyon.

[5] Liu D., Auguste D.T. (2015). Cancer targeted therapeutics: From molecules to drug delivery vehicles. J. Control. Release.

[6] Imtiaz S., Ferdous U.T., Nizela A., Hasan A., Shakoor A., Zia A.W., Uddin S. (2025). Mechanistic study of cancer drug delivery: Current techniques, limitations, and future prospects. Eur. J. Med. Chem..

[7] Feng S., Hu W., Pei F., Liu Z., Du B., Mu X., Liu B., Hao Q., Lei W., Tong Z. (2022). A Highly Sensitive Fluorescence and Screen-Printed Electrodes—Electrochemiluminescence Immunosensor for Ricin Detection Based on CdSe/ZnS QDs with Dual Signal. Toxins.

[8] Nikazar S., Sivasankarapillai V.S., Rahdar A., Gasmi S., Anumol P.S., Shanavas M.S. (2020). Revisiting the cytotoxicity of quantum dots: An in-depth overview. Biophys. Rev..

[9] Lin X., Chen T. (2023). A Review of in vivo Toxicity of Quantum Dots in Animal Models. Int. J. Nanomed..

[10] Le N., Chand A., Braun E., Keyes C., Wu Q., Kim K. (2023). Interactions between Quantum Dots and G-Actin. Int. J. Mol. Sci..

[11] Chand A., Le N., Kim K. (2024). CdSe/ZnS Quantum Dots’ Impact on In Vitro Actin Dynamics. Int. J. Mol. Sci..

[12] Svitkina T. (2018). The Actin Cytoskeleton and Actin-Based Motility. Cold Spring Harb. Perspect. Biol..

[13] Lartey N.L., Schnoor M. (2022). Cellular substructures, actin dynamics, and actin-binding proteins regulating cell migration. Cell Movement in Health and Disease.

[14] Rajan S., Kudryashov D.S., Reisler E. (2023). Actin Bundles Dynamics and Architecture. Biomolecules.

[15] Noureddine M., Mikolajek H., Morgan N.V., Denning C., Loughna S., Gehmlich K., Mohammed F. (2025). Structural and functional insights into α-actinin isoforms and their implications in cardiovascular disease. J. Gen. Physiol..

[16] Chen Y., Barkley M.D. (1998). Toward Understanding Tryptophan Fluorescence in Proteins†. Biochemistry.

[17] Ghisaidoobe A.B.T., Chung S.J. (2014). Intrinsic Tryptophan Fluorescence in the Detection and Analysis of Proteins: A Focus on Förster Resonance Energy Transfer Techniques. Int. J. Mol. Sci..

[18] Weiss J.N. (1997). The Hill equation revisited: Uses and misuses. FASEB J..

[19] Rosenberg S., Stracher A. (1982). Effect of actin-binding protein on the sedimentation properties of actin. J. Cell Biol..

[20] Lin S.S., Chuang M.C., Liu Y.W. (2019). F-actin Bundle Sedimentation Assay. Bio Protoc..

[21] Schmoller K.M., Semmrich C., Bausch A.R. (2011). Slow down of actin depolymerization by cross-linking molecules. J. Struct. Biol..

[22] Jumper J., Evans R., Pritzel A., Green T., Figurnov M., Ronneberger O., Tunyasuvunakool K., Bates R., Žídek A., Potapenko A. (2021). Highly accurate protein structure prediction with AlphaFold. Nature.

[23] Shams H., Golji J., Garakani K., Mofrad M.R.K. (2016). Dynamic Regulation of α-Actinin’s Calponin Homology Domains on F-Actin. Biophys. J..

[24] Tang J., Taylor D.W., Taylor K.A. (2001). The three-dimensional structure of α-actinin obtained by cryoelectron microscopy suggests a model for Ca2+-dependent actin binding. J. Mol. Biol..

[25] Dominguez R., Holmes K.C. (2011). Actin Structure and Function. Annu. Rev. Biophys..

[26] Cano M.L., Cassimeris L., Fechheimer M., Zigmond S.H. (1992). Mechanisms responsible for F-actin stabilization after lysis of polymorphonuclear leukocytes. J. Cell Biol..

[27] Zigmond S.H., Furukawa R., Fechheimer M. (1992). Inhibition of actin filament depolymerization by the Dictyostelium 30,000-D actin-bundling protein. J. Cell Biol..

[28] Lebart M.C., Hubert F., Boiteau C., Ventéo S., Roustan C., Benyamin Y. (2004). Biochemical Characterization of the L-Plastin−Actin Interaction Shows a Resemblance with That of α-Actinin and Allows a Distinction to be Made between the Two Actin-Binding Domains of the Molecule†. Biochemistry.

[29] Chikireddy J., Lengagne L., Le Borgne R., Durieu C., Wioland H., Romet-Lemonne G., Jégou A. (2024). Fascin-induced bundling protects actin filaments from disassembly by cofilin. J. Cell Biol..

[30] McFarlane M., Hall N., McConnell G. (2022). Enhanced fluorescence from semiconductor quantum dot-labelled cells excited at 280 nm. Methods Appl. Fluoresc..

[31] Namsani S., Nair N.N., Singh J.K. (2015). Interaction potential models for bulk Zns, Zns nanoparticle, and Zns nanoparticle-PMMA from first-principles. J. Comput. Chem..

[32] Rahmani R., Lyubartsev A.P. (2023). Biomolecular Adsorprion at ZnS Nanomaterials: A Molecular Dynamics Simulation Study of the Adsorption Preferences, Effects of the Surface Curvature and Coating. Nanomaterials.

[33] Abraham M.J., Murtola T., Schulz R., Páll S., Smith J.C., Hess B., Lindahl E. (2015). GROMACS: High performance molecular simulations through multi-level parallelism from laptops to supercomputers. SoftwareX.

[34] Lindorff-Larsen K., Piana S., Palmo K., Maragakis P., Klepeis J.L., Dror R.O., Shaw D.E. (2010). Improved side-chain torsion potentials for the Amber ff99SB protein force field. Proteins.

